# Epigenetic Profiles Reveal That ADCYAP1 Serves as Key Molecule in Gestational Diabetes Mellitus

**DOI:** 10.1155/2019/6936175

**Published:** 2019-08-14

**Authors:** Xue Li, Wenhong Yang, Yanning Fang

**Affiliations:** ^1^Department of Obstetrics, First People's Hospital of Jining, Jining, Shandong 272000, China; ^2^Department of Nursing, First People's Hospital of Jining, Jining, Shandong 272000, China

## Abstract

Gestational diabetes mellitus (GDM) refers to the condition which shows abnormal glucose metabolism that occurs during pregnancy, while normal glucose metabolism before pregnancy. In the present study, a novel analytical procedure was used to explore the key molecule of gestational diabetes mellitus. First, the weighted pathway model was carried out subsequently to eliminate the gene-overlapping effects among pathways. Second, we assessed the enriched pathways by a combination of Fisher's *t*-test and the Mann–Whitney *U* test. We carried out the functional principal component analysis by estimating *F* values of genes to identify the hub genes in the enriched pathways. Results showed that a total of 4 differential pathways were enriched. The key pathway was considered as the insulin secretion pathway. *F* values of each gene in the key pathway were calculated. Three hub molecules were identified as hub differentially methylated genes, namely, *CAMK2B*, *ADCYAP1*, and *KCNN2*. In addition, by further comparing the gene expression data in a validation cohort, one key molecule was obtained, *ADCYAP1*. Therefore, *ADCYAP1* may serve as a potential target for the treatment of GDM.

## 1. Introduction

Gestational diabetes mellitus (GDM) is the most common medical complication of pregnancy, characterized by glucose intolerance which did not occur before pregnancy but becomes clinically apparent in the late stage of fetation [1]. Glucose metabolism in most GDM patients returns to normal after delivery, but there is an increased chance of developing type 2 diabetes, metabolic syndrome, and cerebrovascular disease in the future [2]. High blood sugar in the womb environment is also very unfavorable to the fetus, increasing the risk of macrosomia and premature delivery and abortion rates, fetal growth restriction (FGR), conferring a predisposition for obesity, neonatal respiratory distress syndrome, cardiovascular complications, and neonatal hypoglycemia [1]. The complication caused by GDM is more complicated and harmful to both mothers and their child.

In view of the serious consequences of GDM, more and more researchers are committed to the study of the dynamic changes in pathogenesis and molecular mechanism of GDM. The occurrence of GDM is related to the formation of insulin resistance in patients. It has been reported that the placenta secretes large amounts of anti-insulin hormones during pregnancy [3]. Inflammatory factors such as C-reactive protein, tumor necrosis factor, serum proteins, interleukin, and other chronic inflammatory reactions were involved in the occurrence of GDM [4, 5]. Analysis of gene methylation data could help us to reveal the relationship between methylation and disease [6]. Epigenome analyses showed that the occurrence of GDM is closely related to changes in gene methylation and expression, especially in the human leukocyte antigens [1]. Several recent reports have described that gene methylation and its expression profile are altered in GDM [7–9]. Research studies showed that distinctive high-level expression changes of genes are associated with their lower promoter methylation prior to the onset of GDM [8]. Although associations between GDM and changes to the epigenomic and genomic profiles for genes have been studied, the molecular landscape changes for pathway level during the GDM are still substantially unknown. It has been elucidated recently that activation of mammalian target of rapamycin (mTOR) pathway upregulated functions of insulin secretion and pancreatic *β*-cells proliferation. Diverse regulation of the mTOR pathway is involved in functions of pancreatic *β*-cells as well as the development of the obstetric complications studied [10].

Currently, researchers use a dynamically modified pipeline, named FUNNEL-GSEA, to analyze the biological mechanisms of gene sets. This kind of model combined the functional principal component analysis and an elastic net regression model [11, 12]. Different from the classic regression analysis, which is a predictive modeling technique that studies the relationship between dependent and independent variables, those techniques were often used for both predictive analysis and causal inference among variables [13]. There are two advances in the methods currently available. First, designed comparisons or regression analyses were not only applied to the comparison between control and experimental groups but also effectively exploited individual information in the group of transcriptomic measurements. Therefore, in this model, one could assess the differences in gene changes among different individuals or time points. Second, it overcomes the problem that overlapping genes, which refers to genes that exist in multiple pathways, play multiple roles in hypothesis testing, where the weight coefficients are overestimated [14].

In our research, we first applied the preweight pathway model to find the enriched signaling pathways of GDM using DNA methylation profiles. Second, we used functional principal component analysis (FPCA) to identify hub genes in the significantly changed pathways. Finally, the gene expression of key molecular pathways in GDM was further tested in an independent cohort.

## 2. Methods

### 2.1. Data Recruitment and Preprocess

DNA methylation data for GDM were deposited in the Gene Expression Omnibus database at GSE70453. A total of 82 sample data were divided into two groups, namely, cases with gestational diabetes and controls without gestational diabetes. The GDM cases contained 41 samples (GDM), and the matched pregnancies (control) contained 41 samples.

A total of 10 GDM patients' tissues and 10 age- and body mass index-matched normal tissues for further validation were obtained from the Department of Obstetrics, First People's Hospital of Jining between June 2016 and June 2017. The decidua basalis placental sampling methods were used as Binder et al. [1]. The umbilical cord was immediately frozen in liquid nitrogen and stored at −80°C after delivery. This study was reviewed and approved by the Ethics Committee of First People's Hospital of Jining (Shandong, China).

### 2.2. Screening for Differentially Methylated Genes

Microarray data contain 473,864 CpG of methylation sites. CpG sites were eliminated when they met the following three types of probes: (1) distance from CpG to single-nucleotide polymorphism (SNP) is less than or equal to 2; (2) minimum equipotential frequency (MAF) less than 0.05; and (3) cross-hybridized probes and probes on sex chromosomes. A total of 426,693 CpGs were kept for further study. In this paper, *β* values represented percentage methylation changes ranging between 0 and 1. Mean *β* values of GDM and normal population were calculated, respectively. We took the mean value of all the related CpGs as the methylation level of a gene since differentially methylated probes may functionally implicate more distal genes. Differentially methylated CpGs were identified at the threshold of *p* < 0.01 and then kept for further study.

### 2.3. Estimating the Weights of Genes in Each Pathway

Genes that present in multiple pathways case the “overlapping problems” which were often overestimated in the enrichment analysis [13]. We basically used the FUNNEL-GSEA model with some modifications to deal with our methylation data [15, 16]. We used the Kyoto Encyclopedia of Genes and Genomes (KEGG) pathway as our database [17]. The weights of the overlapping genes can be obtained by

(1) $$\begin{array}{c} w_{i,k}=\frac{1}{\sum_{k\in k_{i}}\beta_{i}^{k}}, \end{array}$$

where *k*_*i*_ is all the pathways which contain gene *k*. *β* is the vector of gene coefficients, which is set as 1 here. Therefore, the weights of the overlapped genes would be estimated. The model above would decompose an overlapping gene between gene sets and eliminate the effects of overlapping genes [14].

### 2.4. Assessment of Significant Enriched Pathways

Pathway analysis was used to find out significant pathways of the GDM. In this study, Fisher's exact test and the Mann–Whitney *U* (MWU) test were carried out to select enriched pathways. The Mann–Whitney *U* (MWU) test is a rank-based nonparametric test that usually is used in a competitive gene set enrichment analysis. The MWU test utilized the gene weight value to test whether the weight of this gene is significantly greater than other genes of the differential pathway (background genes) [18]. Combined with Fisher's exact test, the final *p* value was calculated as

(2) $$\begin{array}{c} P_{i}=\sqrt[{2}]{P_{\text{MWU}}\times P_{\text{Fisher}}}, \end{array}$$

where *P*_*i*_ is the final *p* value of a pathway *i*, while *P*_MWU_ and *P*_Fisher_ represent the *p* values calculated from the MWU test and Fisher's exact test, respectively. The list of differential methylated genes was assessed as gene list, while the whole genome was set as the background. Pathways with *p* < 0.05 and gene count >1 were extracted and were considered as enriched pathways.

### 2.5. Estimating the *F*-Statistics of Genes in the Enriched Pathways Using the FPCA Model

In this model, each gene gets an *F* value [14]. The mean methylation of each gene is subtracted, and FPCA is adopted across all the centered methylation values. Each gene methylation value is calculated according to the following functions:

(3) $$\begin{array}{c} \hat{X_{i}}\left(t\right)=\hat{\mu_{i}}+\overset{L}{\underset{l=1}{\sum }}\hat{\xi_{il}}\hat{\Phi_{l}}\left(t\right). \end{array}$$

In the above formula, $\hat{\xi_{il}}$ is the FPC score which could quantify how much $\hat{X_{i}}\left(t\right)$ can be explained by $\hat{\Phi_{l}}\left(t\right)$. $\hat{\mu_{i}}$ represents the temporal sample average expression, and $\hat{\Phi_{l}}\left(t\right)$ represents the *l*th eigenfunction.

We net use functional *F*-statistics to summarize the gene pattern information for each gene.

(4) $$\begin{array}{c} F_{i}=\frac{{\text{RSS}}_{i}^{0}-{\text{RSS}}_{i}^{1}}{{\text{RSS}}_{i}^{1}\;+\;\delta }, \end{array}$$

where RSS_*i*_^0^ is the residual sum of squares of null hypotheses, RSS_*i*_^1^ represents the residual sum of squares of alternative hypotheses, *δ* could be considered as a “signal-to-noise” ratio, and *F*_*i*_ revealed the importance of genes [19]. Genes with higher *F* value indicate higher importance.

### 2.6. Identification of Gene Expression of Hub Genes Using Fresh-Frozen Umbilical Cord Tissue by Reverse Transcription-Quantitative Polymerase Chain Reaction (RT-qPCR)

Total RNA was extracted from cells or tissues using TRIzol (Invitrogen; Thermo Fisher Scientific, Inc., Waltham, MA, USA). cDNA synthesis was performed at 37°C for 15 min and then 85°C for 5 sec using reverse transcriptase (Applied Biosystems; Thermo Fisher Scientific, Inc.) following the manufacturer protocol. qPCR was conducted with the ABI 7500 system (Applied Biosystems; Thermo Fisher Scientific, Inc.) using SYBR-Green (Takara Biotechnology Co., Ltd., Jinan, China). PCR was performed for 25 cycles of 10 sec at 98°C, 10 sec at 55°C, and 20 sec at 72°C. The primer sequences used were as follows: CAMK2B: forward, 5′-TACGAGGATATTGGCAAGGG-3′ and reverse, 5′-GCT TCT GGT GAT AGT GTG C-3′; ADCYAP1: forward, 5′-ATC CTT AAC GAG GCC TAC C-3′ and reverse, 5′-CAT TTG TTT CCG GTA GCG G-3′; KCNN2: forward, 5′-CCA GGA ACT GTA CTC TTG GT-3′ and reverse, 5′-ATCATGGTACCTTTCACAAGC-3′; GAPDH: forward, 5′-ACA CCC ACT CCT CCA CCT TT-3′ and reverse, 5′-TTA CTC CTT GGA GGC CAT GT-3′. mRNA expression levels were normalized using GAPDH. Fold changes were counted using the 2-ΔΔCt method.

## 3. Results

### 3.1. Identification of Differentially Methylated Genes

**Figure 1:**
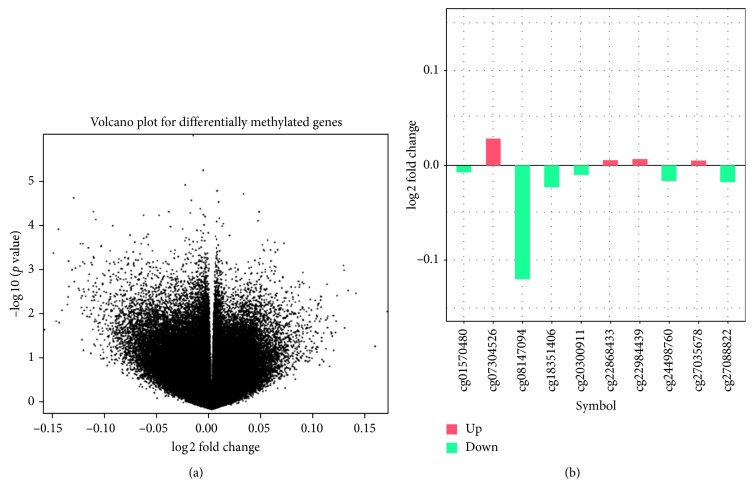
(a) The volcanic map of differentially methylated CpGs. All points in the figure represent all the 426,693 methylated CpGs. The *x*-axis represents methylation differences between GDM and normal (log2-transformed fold change). *Y*-axis was log10-transformed *p* values. (b) The top 10 differentially methylated CpGs.

With the threshold of *p* < 0.01, a total of 2310 differentially methylated CpGs (covering 1520 genes) were obtained. Among the 2310 methylated CpGs, 851 of the CpGs were down-methylated and 1459 of the CpGs were up-methylated in the GDM group.  Figure 1(a) shows the volcanic map of differentially methylated CpGs. According to the threshold, 2310 differentially methylated CpGs initially extracted were subjected to further filtering to obtain the high differentially methylated CpGs. CpGs meeting *S* ≥ 0.1 were retained, resulting in 87 differentially methylated CpGs covered 87 genes. The top 10 differentially methylated CpGs are shown in Figure 1(b).

### 3.2. Screening for Significantly Enriched Pathways Using a Preweighted Pathway Database

**Figure 2:**
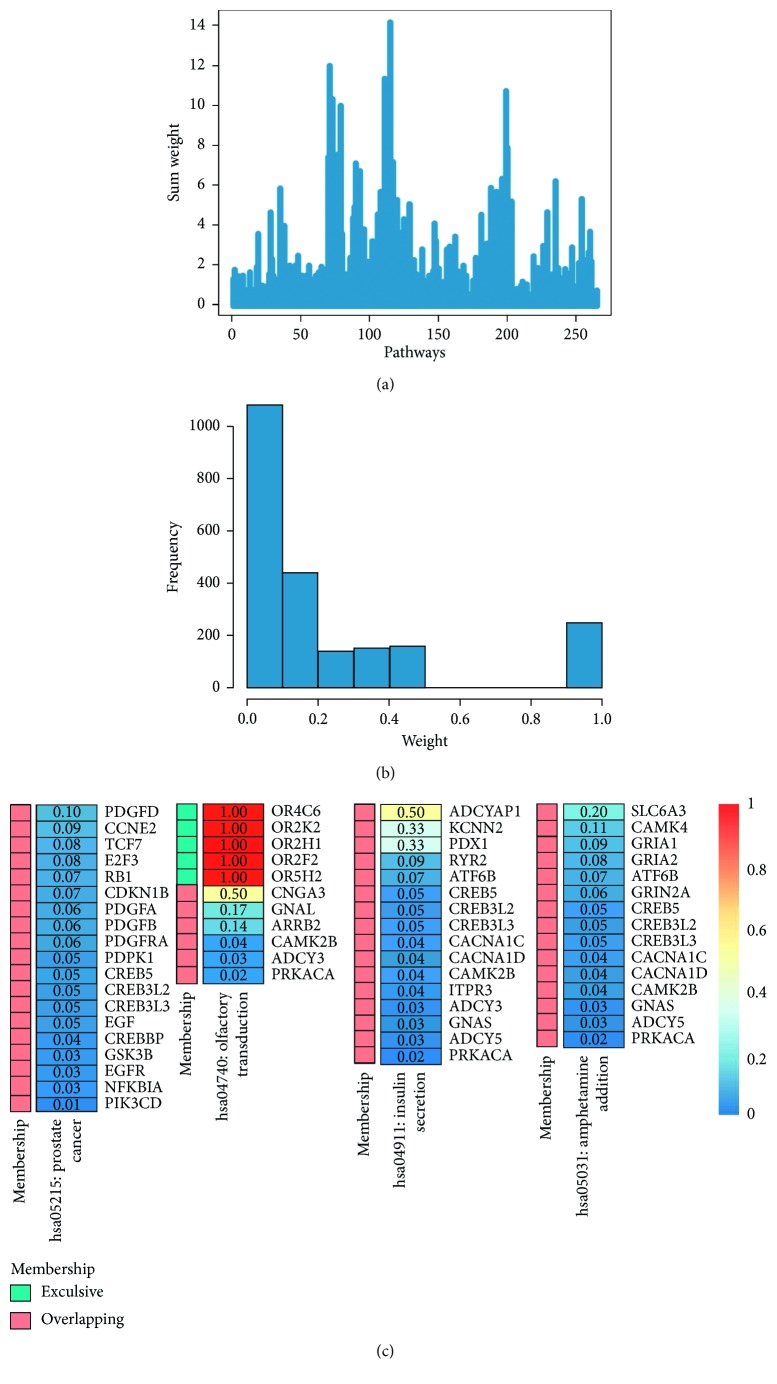
Plot of sum weight (a) and weight distribution of genes (b) for KEGG pathways in GDM and the weights of genes in the 4 enriched pathways (c).

Given a gene associated with multiple gene sets, we assume that the overlapping genes are activated by all gene sets to which they belong. Estimated weights were assigned as 1/*n*, where *n* is the number of gene sets that this gene is associated with. Pathway enrichment analysis of GDM was conducted on the basis of the KEGG pathway database. A total of 286 pathways covered 6893 genes were obtained.  Figure 2(a) shows the distribution of weights of all pathway genes, while Figure 2(b) shows the distribution of sum weights of all pathways and Figure 2(c) shows the weights of genes in the 4 enriched pathways. Based on the preweighted pathway database, 4 differential pathways were yielded.  Table 1 shows the differential signaling pathways in ascending order based on the final *p* value.

**Figure 3:**
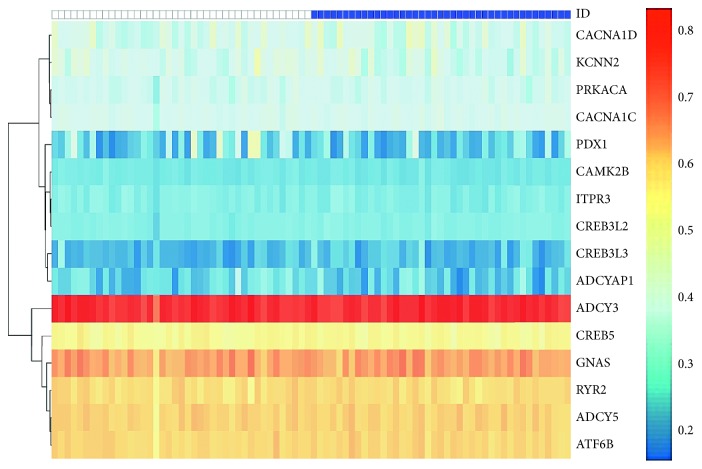
Heatmap of methylation levels of the insulin secretion pathway.

After Fisher's exact test and the Mann–Whitney *U* (MWU) test, 4 members of the pathway are shown in Table 1: olfactory transduction, prostate cancer, insulin secretion, and amphetamine addiction. These 4 signaling pathways may play important roles in the occurrence of GDM. The insulin secretion pathway was considered as the most important pathway and kept for further identification of key molecules in this pathway since it has been widely approved to be associated with GDM [3, 20, 21]. The insulin secretion pathway here contained 16 differentially methylated genes.  Figure 3 shows the heatmap of DNA methylation level of genes in the insulin secretion pathway.

### 3.3. FPCA Analysis of Expression Profile for Hub Genes in the Enriched Pathways

**Figure 4:**
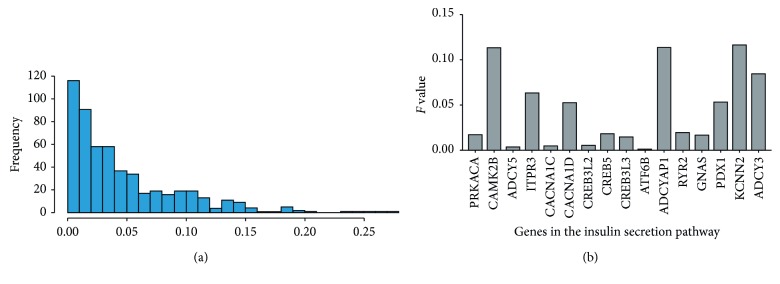
The distribution of *F* value of pathway genes from all pathway genes (a) and insulin secretion pathway (b). Gene methylation data were analyzed by FPCA and each gene got an *F* value (*x*-coordinate, *F* value). *Y*-axis represents gene density.

The FPCA model was used to identify hub genes in the enriched pathways. FPCA could effectively utilize the time series information and overcome the traditional control design deficiencies [14]. Each gene got an *F* value. Higher *F* value indicated a higher activation in their pathways.  Figure 4(a) shows the distribution of the *F* value of all pathway genes.  Figure 4(b) shows the *F* value of all genes in the insulin secretion pathway. Genes and their *F* values were listed in descending order. The top 3 genes (*CAMK2B*, *ADCYAP1*, and *KCNN2*) with high *F* values in the insulin secretion pathway were selected for further validation.

### 3.4. Validation of the Gene Expression of Hub Genes

**Figure 5:**
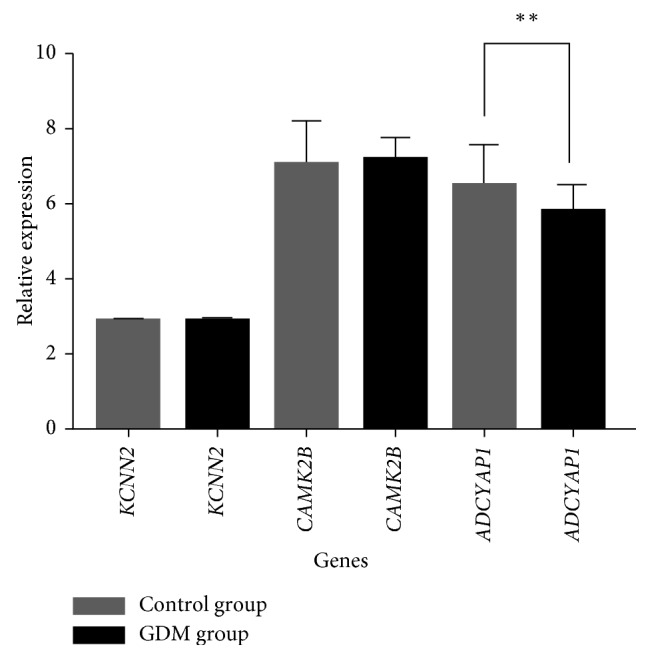
Expression levels of *CAMK2B*, *ADCYAP1*, and *KCNN2*. Grey color represents the control group, and black color represents the GDM group.

To investigate the relationship between the methylation and the gene expression of hub genes in GDM, the additional cohort was used to identify the expressions of hub genes in GDM. The expression profiles of GDM and normal control groups of our cohort were used. By assessing the RNA expression data of hub genes, significant changes between GDM and normal control groups were found in the gene expression of *ADCYAP1*. The expression levels of *CAMK2B*, *ADCYAP1*, and *KCNN2* between GDM and normal control groups are shown in Figure 5.

## 4. Discussion

GDM refers to a varying degree of impaired glucose tolerance occurring for the first time during pregnancy, excluding patients who were with diabetes previous to gestation but were first diagnosed during pregnancy [1]. In China, the incidence of GDM is about 5%–7%, and there is an increasing trend [22]. According to the results of traditional research methods, the etiology of GDM is closely related to insulin resistance. In recent years, with the development of molecular genetics, molecular immunology, and bioinformatics, more and more studies have shown that many factors such as life style, *β*-cell dysfunction, inflammatory factors, and adipokines are involved in the development of GDM [23–25].

Epigenetics refers to heritable changes in gene function that occurs under the condition of not changing the DNA sequence, including DNA methylation, genomic imprinting, maternal effects, gene silencing, and RNA editing [26]. As one of the important epigenetic phenomena, DNA methylation plays an important regulatory role in the gene expression. DNA methylation is closely related to the occurrence and development of many diseases, such as type 2 diabetes [27], autoimmune diseases, and various cancers [28, 29].

In this study, GDM pathogenesis was analyzed using bioinformatics, including KEGG enrichment method, functional principal component analysis (FPCA), elastic net regression, and the Mann–Whitney *U* test. According to this new analytical procedure, four signaling pathways for olfactory transduction, prostate cancer, insulin secretion, and amphetamine addiction were found out. There were some genes involved in the enriched pathways which were related to GDM. Herein, one differentially methylated key molecule was identified: adenylate cyclase activating polypeptide 1 (ADCYAP1). *ADCYAP1* gene encodes a pituitary adenylate cyclase activating polypeptide (PACAP). PACAP is a secreted proprotein with the ability to activate adenylyl cyclase, which is a membrane-bound enzyme that converts ATP to cAMP. ADCY3 is one of the adenylate cyclases that participates in the insulin secretion pathway and also identified as a key molecule/gene by our analysis. Those results provide a novel insight into GDM diagnosis and therapy. Numerous studies demonstrate that PACAP and adenylyl cyclase have a potential role in islet physiology and as a basis for development of islet-promoting therapy in diabetes [30–32]. Adenylyl cyclase is an effector in the G protein-coupled system [33], and its enzymatic activity is under the control of several hormones, including insulin [34, 35]. PACAP and adenylyl cyclase were capable of influencing pancreatic islet function by stimulating pancreatic beta cells to secrete insulin and glucagon [36]. For the clinical treatment of type 2 diabetes, PACAP and adenylyl cyclase were also thought to be effective due to stimulation of insulin secretion [37] and increased proliferation and differentiation of *β* cells [38]. In addition, PACAP is also a neurotransmitter and a member of the vasoactive intestinal peptide/secretin/glucagon peptide superfamily. PACAP shows highly potent neuroprotective and general cytoprotective effects [39]. PACAP is also protective in diabetes-induced pathologies, like retinopathy and nephropathy [40–42]. Consistent with those studies, our results showed that several signal transduction pathways, such as olfactory transduction pathway, were enriched as well.

There are limitations present in our study. Unfortunately, the methylation data along with the expression data for samples were unprovided. Further study will be directly tested for the most significant CpGs that impact upon each hub gene since multiple CpGs could impact the same gene.

## 5. Conclusions

Based on the analytical results of the present study, there was significant *ADCYAP1* methylation and gene expression differences between GDM and normal control groups. GDM was associated with insulin resistance and insulin-signaling system may require *ADCYAP1* participation [43]. We speculate that *ADCYAP1* may be related to the GDM, and more experimental data were needed to support our prediction.

## Figures and Tables

**Table 1:** Significant enriched pathways of GDM.

Pathway name	Fisher's exact test	MWU test	Final *p* value	Count	Total
hsa04740: olfactory transduction	0.0014	0.126083253	0.0013	11	408
hsa05215: prostate cancer	0.0043	0.164116273	0.0026	19	89
hsa04911: insulin secretion	0.0008	0.094356684	0.0091	16	86
hsa05031: amphetamine addiction	0.0001	0.49776561	0.0092	15	68

Count: number of genes in a pathway. Total: total number of genes in a pathway.

## Data Availability

The datasets used and analyzed during the current study are available from the corresponding author on reasonable request.
